# Which population-level interventions promote physical activity equitably across socioeconomic groups? A rapid systematic review

**DOI:** 10.1136/bmjph-2025-004505

**Published:** 2026-05-14

**Authors:** Gemma Frances Spiers, Lisa Mcgarrigle, Tafadzwa Patience Kunonga, Marina Politis, Jabez Barnabas, Fiona Beyer, Jane Mcdermott, Chris Todd, Barbara Hanratty

**Affiliations:** 1 Faculty of Medical Sciences, Newcastle University, Newcastle upon Tyne, UK; 2 Faculty of Biology, Medicine and Health, The University of Manchester, Manchester, UK

**Keywords:** Preventive Medicine, Public Health, Social Medicine, Community Health

## Abstract

**Introduction:**

Promoting physical activity among disadvantaged groups is a major policy goal in many high-income countries. We aimed to summarise evidence on the effectiveness of population-level interventions to promote physical activity among socioeconomically disadvantaged groups.

**Methods:**

A rapid systematic review was undertaken. Searches were conducted in four bibliographic databases in April 2025 (2015–2025) and grey literature, with additional forward citation searching and checking reference lists of relevant reviews. Studies were included if they: evaluated population-level approaches to promoting physical activity in adult populations; compared effectiveness between socioeconomic groups and were published in English from any country. Studies were quality assessed and a narrative synthesis applied.

**Results:**

12 studies (13 publications) were included. Studies were rated moderate (n=3) and low (n=9) in quality. A programme of free access to gym and swim facilities with supplementary health promotion (UK) and a community-wide health promotion programme targeting older adults (Japan) were successful in promoting physical activity among disadvantaged groups while posing a low risk of widening health inequalities. Other interventions identified either did not increase physical activity in disadvantaged groups or posed a high risk of widening health inequalities.

**Conclusion:**

Some population-level approaches are promising, but ongoing monitoring of how these approaches work across different contexts is crucial. Hidden costs must be mitigated against to maximise the benefits of population approaches for the most disadvantaged. There has been modest progress in incorporating equity considerations into evaluations of population-level interventions to promote physical activity.

**PROSPERO registration number:**

CRD420251032624.

**WHAT IS ALREADY KNOWN ON THIS TOPIC**

**WHAT THIS STUDY ADDS**

- Some population approaches to promoting physical activity are promising to promote physical activity among socioeconomically disadvantaged groups while posing a low risk of widening health inequalities.

**HOW THIS STUDY MIGHT AFFECT RESEARCH, PRACTICE OR POLICY**

- The findings suggest there are options for policymakers for promoting equitable physical activity between socioeconomic groups.
- Ongoing evaluation of how these approaches work across different contexts is crucial.

## Background

 Physical activity is critical for good health at all ages. In mid and later life, physical activity brings a range of benefits that delay age-related functional decline and maximise healthy ageing. However, activity levels remain low in adult and older populations and are currently below the levels recommended by the UK Chief Medical Officers and the WHO.^1 2^

Opportunities to engage in physical activity are not equal across populations. Disadvantaged groups face numerous barriers to engaging in regular physical activity, often due to a lack of resources. Costs and poor health are major barriers, while long working hours in manual occupations can leave little time and energy available for exercise.^3^,^5^ Environments also play a role, with greater area deprivation linked to poor accessibility and perceived safety.^6 7^ Yet disadvantaged populations, who are more likely to experience poorer health and earlier onset of disability,^8 9^ are a key group for whom physical activity may offer the largest boost to their health and longevity.

Supporting greater physical activity in disadvantaged groups requires approaches targeting both individuals and populations. Approaches that target individuals have the advantage of responding meaningfully to unique circumstances. Population approaches have the advantage of targeting the social and structural determinants of health and inequalities, with potential widespread benefits across communities.^10 11^ As part of a programme of work to inform policy in the UK, we undertook two linked reviews of evidence, each considering targeted and population approaches to promoting physical activity in disadvantaged groups.

In this paper, we report findings about population-level approaches. Drawing on the classification used by the US Community Preventive Services Taskforce (2021) and Heath *et al*,^12 13^ population approaches include: behavioural and social approaches, informational and campaign approaches, and environmental and policy approaches. Behavioural and social approaches modify social environments or enhance behavioural management skills to enable change in lifestyles in group settings. Information and campaign approaches aim to enhance knowledge and modify attitudes. Environmental and policy approaches aim to improve the accessibility, safety and desirability of physical environments and community infrastructures.

There is growing evidence about population approaches to promoting physical activity, although a review published almost a decade ago found that studies rarely considered the equity of such approaches. More evidence from primary studies has been published since this review, warranting an up-to-date evidence synthesis. This work therefore aimed to:

- Synthesise evidence about the effectiveness of population-level interventions to promote physical activity among disadvantaged groups.
- Synthesise evidence about the acceptability of, and barriers and facilitators to, population-level interventions to promote physical activity among disadvantaged groups.

## Methods

To achieve the aims, a rapid systematic review was undertaken.^14 15^ We chose rapid review methods to offer a timely picture of evidence that could inform concurrent UK policy developments on the topic at the time the work was initiated. Key aspects of the review method that were streamlined for this rapid approach were (a) minor restrictions to the search strategy (limiting the search period, and to English language publications only), (b) applying second checking of extracted data to outcome data only (as opposed to all study information) and (c) using one researcher to undertake risk of bias assessments and a second reviewer to check these (as opposed to having two reviewers independently apply these assessments and appraisals compared). Full methods are reported according to the Preferred Reporting Items for Systematic Reviews and Meta-Analyses (PRISMA).^16^ The protocol was registered on PROSPERO (CRD420251032624).

### Criteria

The PICOS review criteria are summarised in table 1 and explained in detail.

**Table 1:** Review criteria

	Include	Exclude
Population	Interventions targeting adults (18+ years). Populations experiencing socioeconomic disadvantage	Interventions targeted at children (<18 years); or adults and children where data are not reported separately for 18+ populations
Intervention	Any population-level intervention. Population-level interventions must target whole populations (local, regional, national)	
Comparator	Non-disadvantaged groups or no comparator	
Outcome	Any measure of physical activity participation that assesses change in frequency of participation (eg, days per week), duration of physical activity (eg, time spent engaging in activity/time spent sedentary), rates of participation (eg, number of trail path users) or number and type of physical activities participated in. These may be collected through, for example, self- or interview-administered questionnaires (eg, Community Healthy Activities Model Program for Seniors (CHAMPS) or Physical Activity Scale for the Elderly (PASE)), activity diary/log or device (eg, smartwatches; pedometers, accelerometers), administrative datasets and surveys. Any measure of change in intensity of physical activity (eg, Light, Moderate or Vigorous Physical Activity). This may be indicated by Metabolic Equivalents (METs) and related measures such as oxygen consumption (VO2 max), calorie consumption or heart rate. Any process evaluation outcomes (eg, adherence, acceptability, barriers and enablers to intervention engagement and uptake) if reported alongside a physical activity outcome	Outcomes related to physical function (eg, gait speed, muscle function), and physical or mental health outcomes not directly related to exercise intensity
Study design	Any evaluative study design, including randomised and non-randomised approaches. Qualitative data collected as part of a process evaluation are also eligible. Primary studies published from 2015, in English language	Qualitative data not linked to an evaluation of an intervention

*Population*: Interventions for any adult age population were eligible. Interventions targeted solely at children (<18 years) were excluded. A key focus of this review was the effectiveness of interventions for disadvantaged communities. We focused solely on populations experiencing socioeconomic disadvantage. While there are numerous other dimensions of disadvantage (eg, based on gender, ethnicity),^17^ it was not possible to consider all of these within this rapid review. Our pre-review scoping also indicated that operationalisation of disadvantage in evaluations of population-level interventions is typically based on a measure of socioeconomic status. We thus focused on socioeconomic status as the dimension where there is the most evidence, and which will likely have the greatest impact on policy relating to both physical activity and health inequalities. Eligible measures of socioeconomic disadvantage included those measuring individual disadvantage (eg, occupational status, income, education) and area-level measures (eg, area deprivation, proportion of population within an area living under a poverty threshold).

We included studies that compared the effectiveness of the intervention between socioeconomic groups (eg, using subgroup, moderator analyses or stratification by a measure of socioeconomic status).

*Intervention*: Eligible interventions were population-level approaches targeting local, regional or national populations.^10^ We adopted the intervention classification used by the US Community Preventive Services Taskforce (2021) and Heath *et al*^12 13^: behavioural and social approaches, informational and campaign approaches, and environmental and policy approaches. Eligible interventions did not need to be designed with the express aim of promoting physical activity. However, studies must have evaluated a physical activity outcome for inclusion in the review. Interventions that included both population-level and individual-level components, and where data were not reported solely for the population component, were excluded.

*Comparator*: Eligible comparators were historical or geographical control groups, or before and after designs with no control group.

*Outcomes*: Eligible outcomes were measures of physical activity frequency, duration or intensity, rates of participation (including proxies, such as number of travel passengers) or the number and type of activities undertaken. Qualitative data relating to intervention acceptability and barriers and enablers to engaging with an intervention were also eligible.

*Study design*: We included studies using non-randomised and randomised evaluative designs. Non-randomised designs may include, but are not limited to, cohort studies (eg, with a historical control group), time series and before and after approaches with no control group.^18 19^ Qualitative designs reporting process evaluations were also eligible. A previous systematic review of population and individual level interventions to promote physical activity used a search period of 2005–2015.^20^ This review is of high quality and is comparable to our approach with a focus on physical activity promotion and the equity of interventions on a number of dimensions of disadvantage. Key methodological divergences between this previous review and ours reported here are that Lehne and Bolte^20^ review considered (a) both population and individual-level interventions and (b) disadvantage on a number of PROGRESS+ factors. To avoid duplicating this work, we included primary studies dated from 2015.

### Search strategy

A search strategy was developed, piloted and refined by one information specialist and reviewed by a second. Experts in public health were consulted to ask for examples of interventions and terminology to inform the search strategy. Searches were conducted in Medline (OVID), Embase (OVID), CINAHL and TroPHI (Trials Register of Promoting Health Interventions, EPPI-Centre) in April 2025 (2015–2025), and restricted to English language. We searched sources of grey literature, and undertook forward citation searching, and screened the reference lists of two relevant systematic reviews.^20 21^ The search strategy applied to Medline, and all sources of grey literature searched, are in online supplemental materials.

### Study selection

Titles and abstracts were screened for relevance, and the full texts of selected records were retrieved and assessed against the review criteria. For both stages, all screening was undertaken independently by two reviewers, with disagreements resolved through discussion. All records were manually screened in Rayyan, an online platform to manage reviews.^22^

### Risk of bias

Risk of bias for quantitative studies was assessed using the NIH quality assessment tools for the appropriate design.^23 24^ These tools are not designed to provide a total score or rating. However, we applied our own rating system to be able to distinguish the poorer quality studies.

High-quality studies were: randomised controlled trials with adequate blinding and concealment and no major limitations; controlled before and after studies that use appropriate analyses which adjust for relevant confounders, include sensitivity analyses and robustness checks to support judgements on causation, and have no other substantial limitations to study design.

Moderate quality studies were: randomised controlled trials with some limitations; controlled before and after designs that use appropriate analyses which adjust for relevant confounders, include sensitivity analyses and robustness checks to support judgements on causation, but which include limitations to other aspects of study design, such as substantial attrition at follow-up or unclear outcome measurement.

Low-quality studies were: randomised designs with major limitations; controlled before and after studies with no appropriate analyses (eg, descriptive reporting of results) or analyses that do not adjust for relevant confounders, and which have several other design limitations; before and after designs with no control group.

All assessments were checked by a second reviewer.

### Data extraction

A data extraction form was developed using Excel, and piloted and refined. Summary study information (eg, study design, country, measures of socioeconomic status, outcome measures) and outcome data (physical activity outcomes by levels of socioeconomic status) were extracted by single reviewers. All outcome data were checked by a second reviewer.

### Synthesis

A narrative synthesis was used to summarise evidence of effectiveness and risks to health inequalities, grouped by the type of intervention and considering the direction and size of effect (where reported).^25^ To explore whether effectiveness was patterned by levels of socioeconomic disadvantage, we explored data by the socioeconomic status measure used in the study, noting where benefits were greater for the most advantaged groups. Finally, we applied the Demands for Population Health Interventions (DEPTH) framework, which classifies the agentic demand of population-level interventions.^26^ We applied the framework to the interventions identified in our review as a descriptive aid.

### Patient and public involvement

Patients and the public were not involved in the design or conduct of this review due to the rapid nature of the work.

### Findings

**Figure 1:**
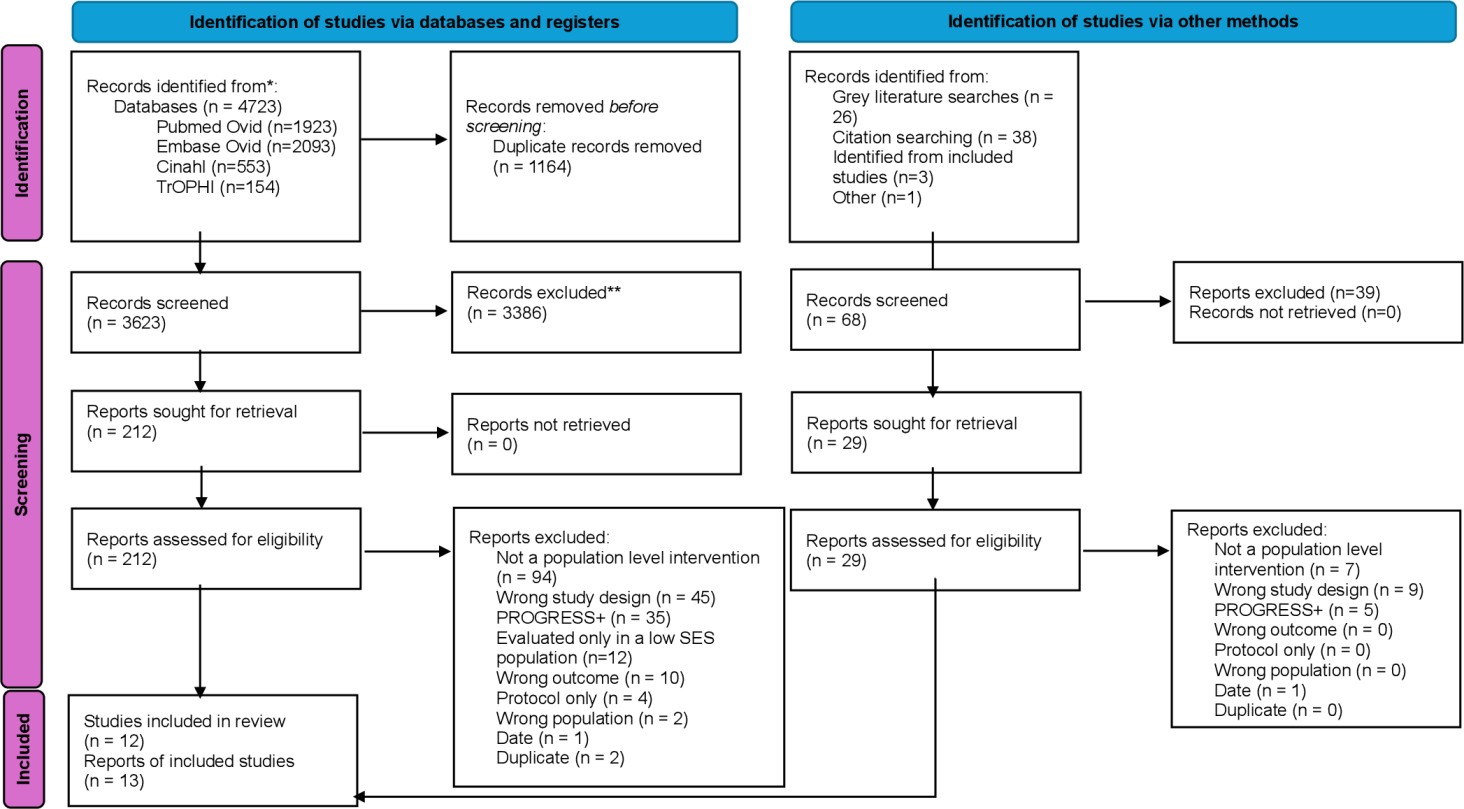
Preferred Reporting Items for Systematic Reviews and Meta-Analyses (PRISMA) flowchart. SES, socioeconomic status.

After screening, 12 studies (reported in 13 publications) were included in the review (figure 1, table 2).^27^,^39^ Studies were from the UK (n=6),^27 28 30 32 33 36 39^ Japan (n=2),^35 37^ Australia (n=2),^34 38^ Germany (n=1)^29^ and the USA (n=1).^31^

**Table 2:** Summary of included studies

Author, country	Intervention	PA outcome(s)	Study design	Measure(s) of socioeconomic disadvantage	Data source	Sample size	Follow-up
*Moderate quality studies*							
Dallmeyer, Breuer^29^ Germany	Introduction of minimum wage	PA frequency Weekly PA frequency	Controlled before and after	Occupational status Educational attainment	German Socio-Economic Panel Survey	2 258 (before), 2 448 (after)	2–4 years
Patterson *et al*^33^ UK	Cycling infrastructure	Cycling commuting prevalence Cycling uptake Cycling maintenance	Controlled before and after	Educational level IMD	Office for National Statistics Longitudinal Survey	25 747	10 years (2001–2011)
Higgerson *et al*^30^ UK	Free access to leisure centres (Re:Fresh)	Gym and swim attendances, gym/swim participation, participation in any moderate-intensity sport or recreation	Controlled before and after	National Statistics Socioeconomic Classification (NS-SEC)	Leisure Management System and Active People Survey (APS)	6 160 (intervention), 1 550 403 (control)	10 years (2005–2015)
*Low-quality studies*							
Candio *et al*^28 48^ UK	Leeds Let’s Get Active. Free universal access to off-peak exercise classes to all city residents	PA frequency, population reach	Non-controlled before and after	IMD	Primary data collection	51 874	Not reported
Panter *et al*^32^ UK	Walking and cycling routes (Connect2)	Weekly time spent walking Time spent walking for recreation	Non-controlled before and after	Educational level, annual household income, employment status	Primary data collection	1 304	1 and 2 years
Le Gouais *et al*^39^ UK	Walking and cycling routes (Connect2)	Pre-post change in % of path users Probability of 50% increase in number of route users	Non-controlled before and after	Area deprivation	Primary data collection	pre=189 250, post=319 531	4 years
Saito *et al*^35^ Japan	Community wide health promotion	Daily duration of PA	Non-controlled before and after	Perceived household economic status	Primary data collection	1 411	2 and 5 years
Tsuzuki *et al*^37^ Japan	Community wide health promotion intervention	Prevalence of regular physical activity	Non-controlled before and after	Employment status	Primary data collection	3 718	2 years
Sloman, 2017 UK	Cycling infrastructure	Any cycling in a typical week	Time series	Social class (A, B, C1, C2, D, E)	Primary data collection	9 000	6-year ITS
Rose *et al*^34^ Australia	$56 million investment by Sport Australia into two funding streams, the Participation (all ages) and Better Ageing (BA, over 65 years)	Meeting PA guidelines	Non-controlled before and after	Area deprivation	Primary data collection	4 761	6 months
Lee *et al*^31^ USA	Relocation to a walkable, mixed-use development	Moderate-to-vigorous physical activity (MVPA)	Controlled before and after	Income	Primary data collection	115	12 months
Veitch *et al*^38^ Australia	Park refurbishment	Adult park visitation	Controlled before and after	Low SES (not otherwise specified) Education Employment	Primary data collection	Not reported	12 and 24 months

IMD, Index of Multiple Deprivation; ITS, interrupted time series; PA, physical activity; SES, socioeconomic status.

Table 3 summarises the interventions that were evaluated. Interventions were improvements to cycling and/or walking infrastructure (n=4), improvements to the environment and green space, such as park refurbishments (n=2), community-wide health promotion campaigns (n=2), free access to leisure facilities (n=2) and investment in national sports programmes (n=1). A final study (n=1) reported evidence about the introduction of the minimum wage. This intervention was not designed to promote physical activity, but the impact on such behaviour was evaluated.

**Table 3:** Summary of interventions

Author	Date	Target population	Intervention summary	Population-level intervention classification^12 13^		
				Behavioural and social approaches	Informational and campaign approaches	Environmental and policy approaches
Dallmeyer, Breuer^29^	2024	All employed adults in Germany	Introduction of minimum wage			☑
Candio *et al*^28 48^	2022, 2020	Adults in Leeds	Leeds Let’s Get Active. Free universal access to off-peak exercise classes to all city residents. Free sessions available to all residents but were provided in leisure centres located in the most deprived areas of the city. Mass media campaign to advertise the offer in 6 months prior		☑	☑
Panter, Ogilvie^32^ Le Gouais *et al*^39^	2017 2021	Adults in regions of the UK	Introduction of Connect2, a programme to make local walking and cycling journeys easier by constructing or improving routes at sites around the UK			☑
Saito *et al*^35^	2021	Older adults, but evaluated in adults aged >20 years in Fujisawa city in Japan	Community-wide health promotion intervention, comprised of delivering information about national PA guidelines and supporting communities to set up their own PA programmes	☑	☑	
Tsuzuki *et al*^37^	2024	All adults in Unnan City aged 40–79 years	Community-wide health promotion intervention, comprising (a) city-wide information delivery (cable television and city newsletters), (b) citywide initiative of a peer-led muscle-strengthening programme, the Unnan Kou-un (Happiness in Unnan) Exercise (promoted through the city-wide leaflets), (c) trained volunteers as community exercise leaders in all communities, (d) restoration of existing swimming pool facility open to all residents in the city	☑	☑	☑
Patterson *et al*^33^ Sloman	2023 2017	All residents in cycling demonstration towns in the UK	Cycling City and Towns Demonstration Programme. Involved improvements in cycle infrastructure; development of town-wide signed networks of cycle routes; branding and marketing of those routes; work with employers, universities, schools and other organisations to help them encourage cycling and improve facilities for cycling at their premises. Cycling Demonstration Towns (CDT) programme ran from October 2005 to March 2011, and involved six medium-sized towns. The Cycling City and Towns (CCT) programme ran from July 2008 to March 2011, and involved one city, one small town and ten medium-sized towns		☑	☑
Rose *et al*^34^	2022	All residents (Participation funding) and over 65 years (Better Ageing)	$56 million investment by Sport Australia into two funding streams, the Participation (all ages) and Better Ageing (BA, over 65 years), to support creation and implementation of programmes in various localities			☑
Lee *et al*^31^	2023	Adults moving into Mueller, a planned, walkable neighbourhood in Austin, Texas	Relocation to a walkable, mixed-use development featuring environmental attributes that support lifestyle physical activity. Higher density and land-use mix, complete sidewalk coverage, a network of parks and trails, 20% of land as public open spaces, 5 miles of hiking and biking trails			☑
Veitch *et al*^38^	2018	Residents in high deprivation neighbourhood	Park refurbishment, main component of which was introduction of a playscape for children			☑
Higgerson *et al*^30^	2018	All residents, workers and GP-registered individuals aged 16 and over in Blackburn with Darwen	Core intervention is universal access to gym and swimming facilities for adults (16+) in Blackburn with Darwen. Supporting components: Health trainer outreach—behavioural support for inactive adults Healthy communities partnership—taster sessions, buddy schemes and community events Marketing and promotion—local media and publicity materials to raise awareness	☑	☑	☑

GP, general practitioner; PA, physical activity.

Three studies were rated moderate in quality^29 30 33^ and nine low in quality (see online supplemental materials).^2831 32 34^,^39^ Moderate-rated studies used controlled before and after designs with strong analytical approaches that adjusted for relevant confounders and undertook robustness and sensitivity checks. The moderate rating for these studies was applied due to other minor study limitations that may introduce some degree of bias. Low-rated studies used either pre-post evaluation designs with no control group or controlled before and after designs with critical limitations to methods.

### Improvements to cycling and/or walking infrastructure

Four studies reported evidence about the impact of improvements to cycling and/or walking infrastructure on physical activity (online supplemental materials table B1).^32 33 36 39^

Two evaluations reported data for the Cycling Demonstration Towns (CDT) and Cycling City and Towns (CCT) programmes in the UK (implemented 2005–2011). Of the two studies, one was rated moderate in quality^33^ and one poor in quality.^32^ The findings summarised next are therefore taken from Patterson *et al*,^33^ which was the strongest study and rated moderate in quality.

The CDT and CCT programmes improved take-up of cycling and walking among both those with (most advantaged) and without (most disadvantaged) a university degree, although the effect size was larger for groups with a degree.^33^ However, take-up of cycling improved for those in areas of high deprivation, but not those living in areas of low deprivation. Overall cycling prevalence did not change as a result of the intervention, for either the most advantaged or disadvantaged groups. Maintenance of cycling among active commuters at baseline was demonstrated for the most advantaged, but not the most disadvantaged.

Change in prevalence of both walking and cycling, or walking alone, did not differ by level of area deprivation. An increase in the prevalence of walking, and both walking and cycling combined, was demonstrated for those without a university degree, but not groups with a university degree. Maintenance of walking, and both walking and cycling combined, for active commuters at baseline improved for both those with and without a degree, but the effect was larger for those with a degree.

Two studies evaluated Connect2, a UK programme to improve cycling and walking routes. Both studies were rated low in quality.^32 39^ In one study, take-up of walking was demonstrated mainly for the most advantaged groups: those with higher levels of education or income.^32^ However, the most disadvantaged (lowest education and income) were more likely to see short-term and sustained increases in time spent walking for transport.^32^ Changes in time spent walking for recreation following the intervention were not patterned on any measure of socioeconomic status. No cycling-related outcome data by socioeconomic status were reported. In the second evaluation of Connect2, there were no statistically significant changes in the number of route users by quintiles of area deprivation.^39^

### Community-wide health promotion

Two studies evaluated community-wide health promotion interventions in Japan (online supplemental materials table B2).^35 37^ One targeted primarily older adults but was evaluated in adults aged 20 years and over in the city of Fujisawa, and the second targeted adults aged 40–79 years in the city of Unnan. Both studies were rated low in quality.

Both community-wide health promotion interventions improved physical activity among advantaged and disadvantaged groups. However, the Fujisawa intervention demonstrated larger improvements in the most disadvantaged groups, but only for adults aged 65+.^30 35^ The same pattern was not observed for adults aged 20–64 years. For the intervention targeting adults 40–79 years, improvements in physical activity favoured the most advantaged populations.^37^

### National investment in two sports programmes

One low-quality study evaluated the impact of a large-scale national investment in two sports programmes, in Australia (online supplemental materials table B3).^34^ One programme, *Participation*, was intended for all ages, and the second, *Better Ageing*, was intended for populations aged 65 years and over.

The study reported no impact of the *Participation* programme on physical activity for either the most advantaged or disadvantaged groups. An improvement in physical activity was only demonstrated for people living in areas with the second-highest deprivation quartile. The *Better Ageing* programme was associated with a reduction in physical activity, although the reduction was larger for the most disadvantaged.

### Free leisure facilities

Two studies (reported over three publications) evaluated the impact of providing free access to leisure facilities in the UK, both in the North of England (Leeds and Blackburn with Darwen) (online supplemental materials table B4).^27 28 30^ The Blackburn with Darwen intervention included free access to gym and swim facilities for adults, supplemented by health trainer outreach, community and taster events and buddy schemes, and a marketing campaign to promote the programme.^30^ The Leeds-based intervention offered free access to off-peak exercise classes (mostly gym and swim sessions). Sessions were offered to all residents in Leeds, but were provided by leisure centres located in the most deprived areas of the city. The programme was preceded by a 6-month marketing campaign to raise awareness of the free facilities.^27 28^

The Blackburn with Darwen-based intervention was reported in one publication, rated moderate in quality. The Leeds-based intervention was reported in two publications, both of which were rated low in quality. (A further evaluation of the Leeds-based intervention was identified that reported cost outcomes and Quality Adjusted Life Expectancy, which was not eligible for inclusion in this review as it did not report physical activity outcomes.)

The Blackburn with Darwen-based intervention improved swim and gym participation, and participation in moderate intensity physical activity, among the most advantaged and disadvantaged groups. Improvements in these outcomes were larger for the most disadvantaged.^30^

In the Leeds-based intervention, take-up of the free leisure programme was relatively low among the most disadvantaged.^28^ Programme participation drop-off was more likely among those living in the most disadvantaged areas. Descriptive data (with no tests of difference) gave an inconsistent picture of changes in active programme users by area of disadvantage.^27^

### Improvements to green space and environment infrastructures

Two studies reported evaluations of improvements to environmental infrastructures (online supplemental materials table B5). These improvements were (a) a park refurbishment in a low socioeconomic neighbourhood (Although delivered in a low socioeconomic neighbourhood, outcome data were reported stratified by educational level and occupational status.) in Australia; (The Australian study of a park refurbishment was also reported in other publications that were not included in this review because they did not report eligible outcomes by socioeconomic status.) and (b) a planned neighbourhood designed to promote physical activity in Austin, Texas.^31^ Both studies were rated low quality (park refurbishment and planned neighbourhood).^31 38^

The walkable neighbourhood in Texas increased physical activity, but the improvements were larger for the most advantaged groups.^31 38^ There was no consistent pattern of findings about improvements for advantaged versus disadvantaged groups for the park refurbishment in Australia.^38^

### Introduction of minimum wage

One moderate quality study evaluated the impact of the introduction of the minimum wage in Germany on physical activity (online supplemental materials table B6). No improvements in physical activity were demonstrated. A reduction in weekly physical activity was recorded for both those in higher occupations and those with low education attainment.

### Evidence of acceptability, barriers and facilitators

No studies were identified that reported evidence about acceptability, barriers and facilitators for disadvantaged groups in receipt of the interventions evaluated.

Which interventions promote physical activity in disadvantaged populations, without posing risks to widening health inequalities?

Table 4 summarises which interventions increased physical activity in the most disadvantaged, and the risks posed by interventions to widening health inequalities. Just two interventions demonstrated larger benefits for the most disadvantaged: a programme offering free access to gym and swim facilities with supplementary health promotion components, in the UK,^30^ and a community-wide health promotion programme targeting primarily older adults in Japan.^35^ This suggests these interventions pose a low risk of widening health inequalities, although the low quality rating of one study^35^ warrants a cautious interpretation.

**Table 4:** Summary of interventions, effectiveness for most and least advantaged, and risk posed to health inequalities

Study, intervention, country	Increased physical activity in least advantaged?	Did the intervention favour the most or least advantaged?* (*data reported where intervention favoured most or least advantaged, but see* *online supplemental materials* *for full data*)	Potential risk posed by intervention to widening health inequalities?†	Quality rating
Higgerson *et al*^30^; free access to leisure facilities+community health promotion components, UK	Yes	Least advantaged (size of difference varied between two outcomes) *Self-reported gym/swimming (≥30 min in past 4 weeks) greater for routine/manual occupations than managerial occupations by +4.7% (4.4% to 5.0%*) *Self-reported moderate PA (≥30 min on ≥12 days in past 4 weeks) greater for routine/manual occupations than managerial occupations by +3.6% (3.3% to 3.8%*)	Low	Moderate quality
Candio *et al*^28 48^; free access to leisure facilities, UK	Yes	Insufficient analyses to determine	Unable to determine	Low quality
Panter, Ogilvie^32^; walking and cycling routes, UK	Yes	Both, depending on outcome measure (see online supplemental materials table B1)	Unable to determine	Low quality
Patterson *et al*^33^; walking and cycling routes, UK	Yes	Both, depending on outcome measure (see online supplemental materials table B1)	Unable to determine	Moderate quality
Sloman 2017; walking and cycling routes, UK	No	Insufficient analyses to determine	Unable to determine	Low quality
Le Gouais *et al*^39^, walking and cycling routes, UK	No	Insufficient analyses to determine	Unable to determine	Low quality
Saito *et al*^35^; community-wide health promotion (for older adults but evaluated in 20+ year adults), Japan	Yes	Least advantaged *Mean difference of change in PA min/day between groups×economic status (ANOVA interaction) for 65+ over 40.9 min/day, p=0.001*	Low	Low quality
Tsuziki *et al*^37^; community-wide health promotion (40–79 years), Japan	Yes	Most advantaged *Difference in adjusted change (baseline to follow-up) in PA between employed/unemployed) +4.4% (0.09% to 8.6%*)	High	Low quality
Dallmeyer^29^ ; minimum wage, Germany	No	Neither	Unable to determine	Moderate quality
Lee *et al*^31^; planned neighbourhood, USA	Not reported	Most advantaged *Change in daily MVPA for highest income compared with lowest: 13.367 min (p<0.05)*	High	Low quality
Rose *et al*^34^; investment in two sports programmes, Australia	No	Neither, but reduction in physical activity greater for least advantaged on one programme	Potentially high	Low quality
Veitch *et al*^38^; park refurbishment, Australia	Inconsistent findings	Insufficient analyses to determine	Unable to determine	Low quality

* Judged by whether improvements in PA were greater for most or least advantaged.

† High risk=intervention favoured most advantaged, low risk=intervention favoured most disadvantaged.

ANOVA, analysis of variance; MVPA, moderate to vigorous physical activity; PA, physical acitivity.

A further potentially promising intervention is the programme of improvements to cycling and walking routes in the UK.^32 33^ Benefits to the most advantaged and disadvantaged varied depending on the outcome measure. However, walking prevalence^33^ and short-lived and sustained increases in walking for transport^32^ improved more so for the most disadvantaged. In the Patterson study, the larger increase in walking prevalence for the most disadvantaged was considered modest. This suggests there may be some scope for this type of intervention to promote physical activity among the most disadvantaged.

For the remaining interventions, the evidence (a) suggested the intervention risked widening health inequalities or (b) was insufficient to make a judgement.

### DEPTH framework

The DEPTH framework, as applied to the intervention components, is summarised in online supplemental materials. Most of the interventions’ components required intended recipients to notice and respond to the intervention’s core features. Only one intervention component was classed as having passive exposure and passive engagement (introduction of the minimum wage). However, this was not an intervention designed to promote physical activity. Overall, there was no clear pattern as to whether any exposure–engagement combinations were linked to intervention effectiveness.

### Measure of socioeconomic status

When considering outcomes by socioeconomic status measure across all studies, there was no pattern to suggest effectiveness consistently varied on one particular measure(s).

## Discussion

Increasing physical activity is a major policy goal in many high-income countries. Successful approaches are especially needed for disadvantaged groups, who face numerous barriers to engaging in physical activity. In this section, we discuss the most promising approaches identified in this review, the implications for policy, and the strengths and limitations of our review.

Promising population-level approaches identified in this review include free access to leisure facilities coupled with community outreach activities (UK) and community-wide health promotion campaign (Japan). Cycling and walking routes may also be promising to promote walking, but not cycling, among disadvantaged groups.

For these approaches, improvements tended to favour disadvantaged groups, suggesting these interventions may pose a low risk of widening health inequalities. Even so, the generalisability of these interventions to other contexts remains uncertain. For example, similar interventions evaluated in different locations and populations either did not show the same findings (community-wide health promotion for 40–79 years old, Japan), or reported insufficient data to allow a clear judgement of effectiveness for disadvantaged groups (free leisure facilities, Leeds, UK). Where these or similar approaches are adopted, population-level data collection to monitor and evaluate impact is crucial.^40^ Other interventions identified either favoured advantaged groups or had no impact on physical activity. For a small number of studies, the data reported were insufficient to inform a judgement on effectiveness by levels of disadvantage.

The effectiveness of free access to leisure facilities (Blackburn with Darwen, UK) among disadvantaged groups fits with broader evidence about the need to offer low-cost options to facilitate physical activity among low-income populations.^41^ In the context of higher living costs faced by many in high-income countries, mitigating the financial costs of opportunities to engage in physical activity is critical. Leisure centre usage can contribute to around a third of the recommended 150 min of moderate to vigorous physical activity.^42^ Enhancing the accessibility of such facilities would therefore offer a promising option to policymakers.

Previous evidence confirms the value and effectiveness of cycling and walking routes to enhance physical activity (eg,^43 44^). However, the studies in this review suggest that not everyone benefits equally from these approaches. Furthermore, disadvantaged groups may find some types of physical activity easier to adopt than others. Walking for transport favoured disadvantaged groups in one study, whereas walking for recreation did not, and cycling tended to favour the more advantaged across studies. This may reflect time and cost barriers to physical activity faced by disadvantaged groups.^5^ For example, hidden costs associated with the purchase of bicycles, cycle equipment or suitable walking shoes could limit uptake. In contrast, walking for transport may offer opportunities to save on day-to-day travel costs and could incentivise the use of walking routes when implemented. Even so, there was evidence of short and sustained increases in walking among disadvantaged groups in one study, signalling the potential of these approaches. To maximise the benefits of planned investments in cycling and walking infrastructure, mitigations are needed to minimise costs for those with limited financial resources.

For some of the interventions evaluated (cycle and walking routes, green space improvements, walkable neighbourhoods), any resultant physical activity would likely occur outdoors. Important policy considerations for these approaches include the needs of populations experiencing mobility issues and/or for those living with frailty, who may find outdoor exercise challenging^45^ and groups who may have concerns about the safety of outdoor exercise, such as women, older, LGBTQ+ or racially minoritised groups.^46^ Furthermore, people living in high deprivation areas tend to spend less time outdoors.^47^ Population approaches that encourage outdoor activity may therefore need to consider mitigations that ensure outdoor activity opportunities are attractive to all, regardless of area of residence.

A previous review identified important gaps in evidence about the effectiveness of physical activity interventions by levels of socioeconomic disadvantage.^20^ Lehne and Bolte’s equity-focused review identified just three studies that reported effectiveness by a measure of socioeconomic status, all of which were evaluations of individual-level, rather than population-level, interventions. The evidence in our review of population-level approaches suggests there has been modest progress in incorporating equity considerations into evaluations of interventions. Where retrospective evaluations use population surveys, which usually include measures of socioeconomic status, failure to consider effectiveness by levels of advantaged is a missed opportunity. Going forward, it is crucial that evaluations of population-level interventions address socioeconomic equity.

Finally, while the quality of studies was mostly poor, it is important to balance this judgement against a pragmatic perspective on what sort of designs are feasible to evaluate population-level approaches. Randomised designs, using cluster or stepped wedge methods, are possible but bring challenges.^18^ In contrast, retrospective evaluation studies using opportunistic controls and population surveys are likely to offer a more feasible and low-cost evaluative option.^19^ While such study designs lack the rigour of randomised methods, strong analytical approaches supplemented by robustness and sensitivity checks can support causal inference.

### Strengths and limitations

Our rapid review used standard, robust methods and a comprehensive search strategy, including a range of grey literature sources. We limited our searches to 2015–2025 to avoid duplicating a previous review on this topic^20^ and thus ensuring our findings reflect contemporary, up-to-date evidence. Pre-review engagement with a range of public health experts was particularly beneficial to optimise our search strategy. Our grey literature searches were predominantly UK focused, while the bibliographic database searches were limited to the English language. This may have resulted in a UK and European-centric evidence base in this review, with more than half of the included studies from this region. Although not a major shortcoming, this geographical focus may limit the generalisability of the conclusions to other global regions.

We focused on socioeconomic disadvantage for this review. While there are numerous other dimensions of disadvantage (eg, gender, ethnicity, disability),^17^ considering all such dimensions was not feasible. This is an important limitation but it does not undermine the conclusions of our work. Critically, the intersecting nature of socioeconomic disadvantage with gender and ethnicity means that our findings have relevance across diverse populations.

Eligible outcomes related to physical activity and process evaluation outcomes, such as acceptability. Other health-related benefits of the identified interventions, as well as cost-effectiveness outcomes, are therefore missed in this review. For example, we are aware that some of the studies we included had associated publications reporting other (non-eligible) outcomes (eg, Candio *et al*^48^). While these other studies may offer useful insights into impact, our approach offers a focused synthesis on impact on physical activity, reflecting the policy focus of our work.

## Conclusion

Free access to leisure facilities and community-wide health promotion campaigns are promising population approaches to increase physical activity among disadvantaged groups while posing a low risk of widening health inequalities. Ongoing monitoring of how these approaches work across different contexts is crucial. Planned investments in walking and cycling infrastructure must also consider how to mitigate against hidden costs that may limit uptake among disadvantaged groups. In the last decade, there has been modest progress in incorporating equity considerations into evaluations of population-level interventions to promote physical activity.

## Supplementary material

10.1136/bmjph-2025-004505 online supplemental file 1

## Data Availability

All data relevant to the study are included in the article or uploaded as supplementary information.
