# Supplementary material for: Which population-level interventions promote physical activity equitably across socioeconomic groups? A rapid systematic review
Source: BMJ Public Health. 2026 May 14;4(2):e004505. doi: 10.1136/bmjph-2025-004505 (PMC13182490; doi:10.1136/bmjph-2025-004505)
Supplement: online supplemental file 1 [file bmjph-4-2-s001.docx]

**Supplementary materials**

**Medline strategy**

**Database(s): Ovid MEDLINE(R) ALL 1946 to April 15, 2025 – Date of Search 16/04/2025
Search Strategy:**

| **#** | **Searches** | **Results** |
| --- | --- | --- |
| **1** | **((promot* or encourag* or boost* or elevat* or develop* or increas* or improv*) adj3 (physical activit* or exercis* or fitness or biking or cycling or walk* or running or active commut* or commut* or physical health or active transport* or cyclist* or bik* or bicycl* or cyclist or active travel* or physically active or mobility* or swim* or sport* or gym* or physical health or active transport* or human powered transport*)).ti,kf.** | **17345** |
| **2** | **Exercise/ or Sports/ or Swimming/ or Running/ or Jogging/ or Walking/** | **267653** |
| **3** | **1 or 2** | **276915** |
| **4** | **("socially disadvantaged" or "general population" or "Population Level" or "disadvantaged" or "marginali?ed" or "poverty" or "less income" or "low income" or "poor" or "less access" or "low access" or "Low-Income Population" or "Low-Income Populations" or "Inadequate Income" or "Low personal income" or "Disadvantaged Populations" or "Underprivileged" or "Social Marginali?ation*" or "Financially poor" or "Economic deprivation" or "Severe lack of money" or "Living in poverty" or "economic hardship" or "socioeconomic" or bankrupt* or "economic insufficiency" or ghetto* or impoverish* or insolven* or "lack of income" or "lack of money" or "no income" or "no money" or slum or slums or unemploy* or "low ses").kf,tw.** | **1253240** |
| **5** | **exp Socioeconomic Factors/** | **534829** |
| **6** | **4 or 5** | **1643704** |
| **7** | **(population based or population?based or population health or community?based or public health or community care or communit* or local communit* or outreach or local administ* or local authorit* or population level*).kf,tw.** | **1445102** |
| **8** | **exp Health Promotion/ or health education/ or public health/ or population health/ or Community Health Services/** | **270946** |
| **9** | **7 or 8** | **1594651** |
| **10** | **((physical activit* or PA or physical health) adj20 (intervention* or effect* or affect or efficac* or random effect* or random-effect or random-effects or active recreation or active travel* or pedestrian activit* or fall prevention* or mobili* or mobility goal* or health effect* or health?system or health system* or health gain* or inequit* or health equity or equit* or environmental impact* or health effect* or health impact* or policy approach* or active transport* or socioeconomic inequities or socioeconomic inequalities or health disparit* or health inequalities or health promotion* or campaign or media or banner* or "participation rates" or "number of users on cycle paths " or "Take up of cycling" or "Moderate intensity activity" or "Counts of trail users" or "Number of exercise sessions per week" or "active transport to park" or "park visitation" or "leisure facilit*" or "leisure time exercise" or "active transportation" or "number of walks" or "bike trip*" or "self reported walking or cycling" or "Walking for transportation or leisure" or "Walking for transport" or "Number of cyclist*" or mobility limitation* or "Total daily physical activit*" or "Recreational physical activit*" or "Traffic volume of pedestrian*" or "pedestrian count*" or "Traffic volume of cyclist*" or "health inequalitie*" or "Change in walking" or "PA not otherwise specified" or "Walking one hour daily" or "Intention to be active" or "Change in exercise" or "Days a week of VPA" or "self-reported physical activity" or streetscape or self-explaining road* or self explaining road* or neighb?hood or physical environment* or urban environment* or suburban environment* or built environment* or community environment* or travel environment* or street environment* or road environment* or shared space* or traffic calm* or smart growth or new urbanism or urban form or urban design or walkability or residential density or community design or city planning or environment design or urban renewal or sidewalk or footpath or green space* or recreational facilit* or public transit or public transport* or block size* or street connectivity or open space* or woonerf or sustainable safety or cycleway or (cycl* and trail*) or (bik* and trail*) or (bicycle* and trail*) or (walk* and trail*) or (pedestrian* and trail*) or (route and school) or (bik* and (path* or lane*)) or (bicycle* and (path* or lane)) or (cycl* and (path* or lane)))).kf,tw.** | **90545** |
| **11** | **exp clinical trial/ or Treatment Outcome/ or Delivery of Health Care/ or Health Inequities/ or Health Impact Assessment/** | **2166359** |
| **12** | **10 or 11** | **2242819** |
| **13** | **Humans/ not (Animals/ and Humans/)** | **20304359** |
| **14** | **3 and 6 and 9 and 12 and 13** | **3280** |
| **15** | **((child or adolescent) not (adult or elderly)).kf,tw.** | **601755** |
| **16** | **14 not 15** | **2971** |
| **17** | **limit 16 to yr="2015-Current"** | **1923** |

**Grey Literature Sources**

- Association of Directors for Public Health <https://www.adph.org.uk/>
- Royal Society for Public Health <https://www.rsph.org.uk/>
- Northern Ireland Public Health Agency <https://www.publichealth.hscni.net/>
- European Public Health Association <https://eupha.org/>
- DEFRA <https://randd.defra.gov.uk/>
- Sustrans <https://www.sustrans.org.uk/>
- UK Government Social Research & Evaluation page <https://www.gov.uk/government/collections/social-research-and-evaluation#cycling,-wheeling-and-walking>
- Active Travel England <https://www.activetravelengland.gov.uk/>
- Transport Action Network <https://transportactionnetwork.org.uk/>
- Movement for Health <https://www.movementforhealth.scot/>
- Sports England <https://www.sportengland.org/funding-and-campaigns/moving-healthcare-professionals>
- International Society for Physical Activity and Health <https://ispah.org/>
- European Network for Workplace Health Promotion <https://www.enwhp.org/>
- World Health Organization [www.who.int](http://www.who.int)
- Local Government Association [www.local.gov.uk](http://www.local.gov.uk)
- UK Active <https://www.ukactive.com/>
- The King’s Fund Database <https://koha.kingsfund.org.uk/>

**Data summary tables for included studies**

Table B1. Data summary table for interventions improving cycling and walking paths

| **Author** | **Date** | **Outcome** | **SES groups compared** | **Estimate and CIs by SES group** | **p value** |
| --- | --- | --- | --- | --- | --- |
| Panter^22^ | 2017 | Uptake of walking for transport | (Ref: tertiary) Secondary school or higher  Lower than secondary | 1.0 0.50 (0.31 to 0.82)  0.76 (0.45 to 1.29) | p<0.05 |
|  |  | Short lived increase in walking for transport | (Ref: tertiary) Secondary school or higher  Lower than secondary | 1.0 1.61 (0.72 to 3.58)  1.36 (0.54 to 3.44) | p<0.05 |
|  |  | Sustained increase in walking for transport | (Ref: tertiary) Secondary school or higher  Lower than secondary | 1.0 1.15 (0.52 to 2.58)  3.21 (1.48 to 6.94) | p<0.01 |
|  |  | Uptake of walking for recreation | (Ref: tertiary) Secondary school or higher  Lower than secondary | 1.0 0.66 (0.41 to 1.07)  0.34 (0.20 to 0.60) | p<0.001 |
|  |  | Short lived increase in walking for recreation | (Ref: tertiary) Secondary school or higher  Lower than secondary | 1.0 0.59 (0.29 to 1.21)  0.59 (0.26 to 1.31) | NS |
|  |  | Sustained increase in walking for recreation | (Ref: tertiary) Secondary school or higher  Lower than secondary | 1.0 1.20 (0.62 to 2.33) 1.55 (0.73 to 3.28) | NS |
|  |  | Uptake of walking for transport | (Ref: any car)  No car | 1.0 1.24 (0.54 to 2.85) | NS |
|  |  | Short lived increase in walking for transport | (Ref: any car)  No car | 1.0 4.77 (2.42 to 9.42) | p<0.001 |
|  |  | Sustained increase in walking for transport | (Ref: any car)  No car | 1.0 5.35 (2.58 to 11.08) | p<0.001 |
|  |  | Uptake of walking for recreation | (Ref: any car)  No car | 1.0 0.68 (0.37 to 1.25) | NS |
|  |  | Short lived increase in walking for recreation | (Ref: any car)  No car | 1.0 0.51 (0.15 to 1.70) | NS |
|  |  | Sustained increase in walking for recreation | (Ref: any car)  No car | 1.0 1.18 (0.51 to 2.72) | NS |
|  |  | Uptake of walking for transport | (Ref: >40 000)  20 001–40 000 ≤20 000 | 1.0 1.17 (0.71 to 1.93)  0.74 (0.42 to 1.31) | NS |
|  |  | Short lived increase in walking for transport | (Ref: >40 000)  20 001–40 000 ≤20 000 | 1.0 1.92 (0.69 to 5.33)  3.43 (1.26 to 9.33) | p<0.01 |
|  |  | Sustained increase in walking for transport | (Ref: >40 000)  20 001–40 000 ≤20 000 | 1.0 2.41 (1.00 to 5.82)  3.79 (1.55 to 9.26) | p<0.01 |
|  |  | Uptake of walking for recreation | (Ref: >40 000)  20 001–40 000 ≤20 000 | 1.0 0.63 (0.37 to 1.08)  0.49 (0.28 to 0.85) | p<0.05 |
|  |  | Short lived increase in walking for recreation | (Ref: >40 000)  20 001–40 000 ≤20 000 | 1.0 0.59 (0.28 to 1.26)  0.63 (0.29 to 1.40) | NS |
|  |  | Sustained increase in walking for recreation | (Ref: >40 000)  20 001–40 000 ≤20 000 | 1.0 1.11 (0.58 to 2.12)  0.58 (0.25 to 1.33) | NS |
|  |  | Uptake of walking for transport | (Ref: working/student)  Retired Unemployed/other/sick | 1.0 1.10 (0.60 to 2.00)  1.15 (0.46 to 2.85) | NS |
|  |  | Short lived increase in walking for transport | (Ref: working/student)  Retired Unemployed/other/sick | 1.0 2.59 (0.96 to 6.95)  0.84 (0.19 to 3.74) | NS |
|  |  | Sustained increase in walking for transport | (Ref: working/student)  Retired Unemployed/other/sick | 1.0 2.85 (1.10 to 7.40)  1.94 (0.70 to 5.38) | NS |
|  |  | Uptake of walking for recreation | (Ref: working/student)  Retired Unemployed/other/sick | 1.0 0.67 (0.33 to 1.35)  0.74 (0.33 to 1.65) | NS |
|  |  | Short lived increase in walking for recreation | (Ref: working/student)  Retired Unemployed/other/sick | 1.0 1.60 (0.68 to 3.75) 1.05 (0.30 to 3.63) | NS |
|  |  | Sustained increase in walking for recreation | (Ref: working/student)  Retired Unemployed/other/sick | 1.0 1.59 (0.68 to 3.76)  0.21 (0.03 to 1.59) | NS |
| Le Gouais^29^ | 2021 | Change in % of users pre-post | IMD quintile 1 (most deprived)  2  3  4  5 (least deprived) | -0.01  -0.2  1.1  -1.4  0.1 | 0.703  0.956  0.654  0.669  0.731 |
|  |  | Probability of 50% increase in number of route users | Area deprivation (data not reported by level) | Area deprivation was not associated with a 50% increase in route users | NS |
| Sloman^26^ | 2017 | Proportion of respondents reporting any cycling in a typical week in the previous year | Social class AB 2006 Social class AB 2009 Social class AB 2011 | 32% 37% 36% | NR |
|  |  |  | Social class C1 2006 Social class C1 2009 Social class C1 2011 | 28% 31% 32% | NR |
|  |  |  | Social class C2 2006 Social class C2 2009 Social class C2 2011 | 26% 29% 30% | NR |
|  |  |  | Social class DE 2006 Social class DE 2009 Social class DE 2011 | 16% 18% 16% | NR |
| Patterson^23^ | 2023 | Change in cycling prevalence | Low deprivation High deprivation | 0.91 (1.19 to 0.69) 1.06 (1.37 to 0.83) | NR |
|  |  | Change in walking prevalence | Degree No degree | 1.19 (1.53 to 0.93) 1.16 (1.31 to 1.03) | NR |
|  |  | Change in walking prevalence | Low deprivation High deprivation | 1.15 (1.37 to 0.97) 1.10 (1.33 to 0.91) | NR |
|  |  | Change in walking or cycling prevalence | Degree No degree | 1.18 (1.44 to 0.97) 1.13 (1.26 to 1.02) | NR |
|  |  | Change in walking or cycling prevalence | Low deprivation High deprivation | 1.09 (1.26 to 0.93) 1.09 (1.27 to 0.94) | NR |
|  |  | Take up cycling | No degree Degree | 1.31 (1.04, 1.64) 1.84 (1.31, 2.59) | NR |
|  |  | Take up cycling | Low deprivation High deprivation | 1.34 (0.98, 1.82) 1.42 (1.04, 1.95) | NR |
|  |  | Take up walking | No degree Degree | 1.24 (1.08, 1.42) 1.57 (1.20, 2.04) | 0.03 <0.001 |
|  |  | Take up walking | Low deprivation High deprivation | 1.19 (0.98, 1.44) 1.23 (0.99, 1.52) | 0.087 0.056 |
|  |  | Take up walking or cycling | No degree Degree | 1.20 (1.05, 1.36) 1.85 (1.48, 2.31) | 0.006 <0.001 |
|  |  | Take up walking or cycling | Low deprivation High deprivation | 1.24 (1.03, 1.48) 1.25 (1.04, 1.51) | 0.021 0.020 |
|  |  | Maintenance of cycling | No degree Degree | 1.07 (0.76, 1.51) 1.81 (1.01, 3.26) | NR |
|  |  | Maintenance of cycling | Low deprivation High deprivation | 1.17 (0.71, 1.92) 1.26 (0.78, 2.01) | NR |
|  |  | Maintenance of walking | No degree Degree | 1.21 (1.00, 1.48) 1.89 (1.20, 2.96) | 0.053 0.006 |
|  |  | Maintenance of walking | Low deprivation High deprivation | 1.26 (0.96, 1.65) 1.35 (0.96, 1.89) | 0.100 0.080 |
|  |  | Maintenance of walking or cycling | No degree Degree | 1.32 (1.12, 1.56) 1.71 (1.21, 2.41) | 0.001 0.002 |
|  |  | Maintenance of walking or cycling | Low deprivation High deprivation | 1.28 (1.01, 1.61) 1.41 (1.08, 1.84) | 0.039 0.012 |

Table B2. Data summary table for community wide health promotion interventions

| **Author** | **Date** | **Outcome** | **SES groups compared** | **Estimate and CIs** | **p value** |
| --- | --- | --- | --- | --- | --- |
| Saito^25^ | 2021 | Mean difference of change in PA minutes/day between groups x economic status (ANOVA interaction) for 20-64 years | Perceived economic status (poor/very poor v average/good/excellent) | -3.3 mins/day | p=0.657 |
|  |  | Mean difference of change in PA minutes/day between groups x economic status (ANOVA interaction) for 65+ over | Perceived economic status (poor/very poor v average/good/excellent) | 40.9 mins/day | p=0.001 |
| Tsuzuki^27^ | 2024 | Adjusted change in prevalence of regular physical activity from baseline to follow up (%) | Employed Unemployed | +9.5%pt (7.0, 12.1) +5.2%pt (1.9, 8.5) | NR |
|  |  | Difference in adjusted change (baseline to follow up) in PA between employed/unemployed) (%) | NA | +4.4%pt (0.09, 8.6) | <0.05 |

Table B4. Data summary table for free leisure facility interventions

| **Author** | **Date** | **Outcome** | **SES groups compared** | **Estimate and CIs** | **p value** |
| --- | --- | --- | --- | --- | --- |
| Candio^18^ | 2022 | IMD profile of residents reached (proportion of total number of adult residents who signed up to the programme over 39 months of implementation), comparing with Leeds city profile | IMD (top 20% deprived versus remaining 80%) | The programme reached a smaller proportion of those living in the top 20% most deprived areas of the city (19.5% living in top 20% most deprived areas of the city reached by programme, compared to 33% of population living in top 20% most deprived areas of the city) | NR |
| Candio^17^ | 2021 | % of participants inactive at baseline | IMD non-deprived IMD deprived | 28.1 32.8 | NR |
|  |  | % participants inactive post-registration survey | IMD non-deprived IMD deprived | 7.6 5.8 |  |
|  |  | % of participants insufficiently active at baseline | IMD non-deprived IMD deprived | 37.6 34.9 | NR |
|  |  | % participants insufficiently active post-registration survey | IMD non-deprived IMD deprived | 32.7 30.2 |  |
|  |  | % of participants moderately active at baseline | IMD non-deprived IMD deprived | 21.7 20.1 | NR |
|  |  | % participants moderately active post-registration survey | IMD non-deprived IMD deprived | 41.9 46.5 |  |
|  |  | % of participants active at baseline | IMD non-deprived IMD deprived | 12.6 12.2 | NR |
|  |  | % participants active post-registration survey | IMD non-deprived IMD deprived | 17.8 17.4 |  |
| Higgerson^20^* | 2017 | Self-reported gym/swimming (≥30 mins in past 4 weeks) | Routine/manual Intermediate Managerial and Professional | +4.7% ( 4.4 to 5.0)  Data not reported  Data not reported | NR |
|  |  | Self-reported moderate PA (≥30 min on ≥12 days in past 4 weeks) | Routine/manual Intermediate Managerial and Professional | +3.6% (3.3 to 3.8)  Data not reported  Data not reported | NR |

*Data for routine and manual reported in text, data for intermediate and managerial/professional reported in graph only. In the text, the effect for participation in 30 minutes of moderate swim or gym activity for the lowest occupational group is 4.7% (CI 4.4-5.5); the effect for participation in 30 minutes moderate PA on at least 12 days for the lowest occupational group is 3.6% (CI 3.3-3.8). However, the estimates as show in the figure appear to be different - with the 4.7% effect for participation in 30 minutes moderate PA on 12 days, and the 3.6% effect for 30 minutes of moderate swim or gym activity. We have reported the data as detailed in the text (rather than the figure) of the publication. Study authors were contacted for clarification.

Table B5. Data summary table for greenspace and built environment interventions

| **Author** | **Date** | **Outcome** | **SES groups compared** | **Estimate and CIs** | **p value** |
| --- | --- | --- | --- | --- | --- |
| Veitch^28^ | 2018 | % Adult park visitation during study period Intervention T1 | Working full time Working part time Unemployed Retired | 36.8  27.8  22.9  12.5 | NR |
|  |  | % Adult park visitation during study period Intervention T3 | Working full time Working part time Unemployed Retired | 38.9  19.7  23.9  17.5 | NR |
|  |  | % Adult park visitation during study period control T1 | Working full time Working part time Unemployed Retired | 33.6  29.2  23.9  13.3 | NR |
|  |  | % Adult park visitation during study period control T3 | Working full time Working part time Unemployed Retired | 31.2  29.8  20.7  18.4 | NR |
|  |  | % Adult park visitation during study period Intervention T1 | No formal qualifications  Year12/apprentice/diploma University degree/ higher degree | 14.6  34.0  51.4 | NR |
|  |  | % Adult park visitation during study period Intervention T3 | No formal qualifications  Year12/apprentice/diploma University degree/ higher degree | 14.2  35.2  50.6 | NR |
|  |  | % Adult park visitation during study period control T1 | No formal qualifications  Year12/apprentice/diploma University degree/ higher degree | 5.2 25.7  69.1 | NR |
|  |  | % Adult park visitation during study period control T3 | No formal qualifications  Year12/apprentice/diploma University degree/ higher degree | 7.4 20.3  72.3 | NR |
| Lee^21^ | 2023 | Change in daily MVPA (SE) | Income <50k Income 50-100K Income 100k+ | Ref 1.525 (6.019) 13.367* (5.732) | <0.05 |

Table B6. Data summary table for introduction of the minimum wage

| **Author** | **Date** | **Outcome** | **SES groups compared** | **Estimate and CIs** | **p value** |
| --- | --- | --- | --- | --- | --- |
| Dallmeyer^19^ | 2024 | Total PA frequency | Blue collar pre intervention Blue collar post intervention White collar pre intervention White collar post intervention | -0.119 (0.174) -0.206 (0.204) -0.410 (0.157) -0.049 (0.157) | Blue collar=NS White collar=p<0.01 |
|  |  | Weekly PA frequency | Blue collar pre intervention Blue collar post intervention White collar pre intervention White collar post intervention | -0.073 (0.674) -0.108 (0.082) -0.182 (0.060) -0.021 (0.063) | Blue collar=NS White collar=p<0.01 |
|  |  | Total PA frequency | High education pre intervention High education post intervention Low education pre intervention Low education post intervention | -0.253 (0.181) 0.032 (0.242) -0.184 (0.137) 0.011 (0.138) | NS |
|  |  | Weekly PA frequency | High education pre intervention High education post intervention Low education pre intervention Low education post intervention | -0.097 (0.052) -0.031 (0.055) -0.139 (0.068) -0.023 (0.089) | High=p<0.10 Low=p<0.05 |

Table B3. Data summary table for sports investment intervention

| **Author** | **Date** | **Outcome** | **SES groups compared** | **Estimate and CIs** | **p value** |
| --- | --- | --- | --- | --- | --- |
| Rose^24^ | 2022 | Participation funding stream: Adjusted odds of meeting physical activity guidelines between pre and post follow up | Employed Unemployed Student Pension/welfare | 1.07 (0.74, 1.55) 0.39 (0.12, 1.22) 1.26 (0.71, 2.21) 3.29 (1.75, 6.2) | NR |
|  |  | Better ageing funding stream: Adjusted odds of meeting physical activity guidelines between pre and post follow up | Employed Unemployed Student Pension/welfare | 0.76 (0.53, 1.08) 0.39 (0.23, 0.65) NA for student 0.93 (0.51, 1.71) | NR |
|  |  | Participation funding stream: Adjusted odds of meeting physical activity guidelines between pre and post follow up | SEIFA 1 (most disadvantaged) SEIFA 2 SEIFA 3 SEIFA 4 (least disadvantaged) | 1.16 (0.72, 1.88) 1.96 (1.10, 3.50) 1.01 (0.59, 1.70) 1.06 (0.63, 1.78) | NR |
|  |  | Better ageing funding stream: Adjusted odds of meeting physical activity guidelines between pre and post follow up | SEIFA 1 (most disadvantaged) SEIFA 2 SEIFA 3 SEIFA 4 (least disadvantaged) | 0.31 (0.17, 0.56) 0.68 (0.47, 0.98) 0.90 (0.67, 1.22) 0.59 (0.45, 0.76) | NR |

**Components of included interventions classified by the DEPTH Framework^16^**

| **Exposure** | **Engagement** | **Mechanism(s) of action** | | | | |
| --- | --- | --- | --- | --- | --- | --- |
|  |  | **Socio-cultural** | **Cognitive** | **Financial** | **Physical environment** | **Biomedical** |
| Active | Active | Supporting communities to create their own PA programmes^25^  City-wide muscle strengthening programme^27^  Working with local organisations to support cycling and promote awareness of routes^23,26,32^ | Lectures in local organisations^25^ | Universal leisure facilities^17,18,20^ | Cycling/walking routes^22,23,26,29,32^  Park refurbishment^28^  Planned, walkable neighbourhood^21^  Swimming pool restored and available to all residents^27^ |  |
|  | Passive |  |  |  |  |  |
| Passive | Active |  | Marketing campaign to raise awareness of intervention^23,25-27,32^  Disseminating PA-related information/health promotion^25,27^ |  |  |  |
|  | Passive |  |  | Introduction of minimum wage^19^ |  |  |

We were unable to classify the National Investment Programme (Rose et al., 2022), due to the absence of detail of the sports programmes that resulted from the investment.

Quality assessment of included studies

| **Author, date** | **Summary of appraisal** | **Rating** |
| --- | --- | --- |
| Sloman, 2017  UK | A before and after study using a representative cohort survey. Major limitation is that the data of interest for this review (PA outcome by SES) is descriptive and was not subject to robust analysis. | Low |
| Patterson, 2023  UK | Controlled before and after study with strong DiD analysis, adjusting for a range of confounders and with robustness checks. Overall, this is a well conducted study, but with the limitations of a non-randomised design and the absence of blinding. There is a lack of reporting around attrition of data at follow up of linked data, giving some uncertainty around this as a potential source of bias. Another limitation is that the intervention allocation was not coterminous with the local authority areas used to identify participants. Authors suggest there may be some residual error measurement, although sensitivity analyses indicates consistency of findings. There is a lack of detail about potential contamination of other interventions occurring in the intervention sites. | Moderate |
| Panter, 2017  UK | A before and after study with some important limitations regarding representativeness of the sample and loss to follow up. It is unclear if other local changes took place during the intervention at community level that would have impacted the outcome measures, which the authors acknowledge. | Low |
| Le Gouais, 2021  UK | A before and after study with some minor limitations around lack of clarity on eligible participants. It is unclear if other local changes took place during the intervention at community level that would have impacted the outcome measures. | Low |
| Higgerson, 2017  UK | Controlled before and after study with strong DiD analysis, adjusting for a range of confounders and performing sensitivity analyses on the SES stratified analysis (by removing 'never worked').  Outcome measures may be subject to bias due to potentially missing swipe card data, or due to self-report, although the authors compared PA levels in the survey with other survey data (HSE) and found comparable levels. There is a lack of detail about potential contamination of other interventions occurring in the intervention sites. The authors note that they were not able to distinguish the effect of the media campaign from the free leisure centre access, so it is not possible to ascertain which (or if both) contributed most to the positive impact on PA. Follow up data rates are not reported, which is an important omission. Whilst reporting quality is generally good, data for the middle and higher categories of NSSEC are not explicitly reported, except as a figure (author contacted for clarification). Finally, authors note that 7.4% of SES data was excluded due to not being classifiable, which may lead to skewed estimates, although by how much is uncertain. | Moderate |
| Candio, 2022, 2020  UK | 2022: The data of relevance to this review is a comparison of reach and programme participation drop off between two IMD profiles, without before and after comparison. Only descriptive data are available for reach, with no analysis of difference. Appropriate test of difference used for programme drop off. Other important sources of bias include sample representativeness.  2020: A before and after study. The data relevant to this review are descriptive and not subject to robust analysis. There are other important limitations including substantial differences in study population between initial registration data and post survey data, and a lack of clarity about the representativeness of the data. | Low |
| Dallmeyer, 2024  Germany | Controlled before and after study with strong DiD analysis, adjusting for a range of confounders and with robustness checks. Issues relating to adherence and presence of other confounding schemes not applicable as this was a national policy directly impacting certain people. Potential important biases may include an outcome measure that was too broad to detect changes in intensity of PA. | Moderate |
| Saito, 2021  Japan | A before and after study with some important limitations regarding representativeness of the sample, and the absence of a baseline measure (with change assessed between 2 and 5 years after the intervention introduced). It is unclear if other local changes took place during the intervention at community level that would have impacted the outcome measure. Other aspects of the study were not reported, with some potential risks of bias unclear. | Low |
| Tsuzuki, 2024  Japan | A before and after study with the associated limitations, including a potentially non-representative sample. Attrition bordered on 20%, but further analysis indicated this may be unlikely to introduce substantial bias. | Low |
| Lee, 2023  US | A controlled before and after study using DiD analysis adjusting for relevant covariates, but with significant limitations in terms of small sample size, no reporting of attrition, or numbers excluded after matching. | Low |
| Veitch, 2018  Australia | A controlled before and after study, a major limitation of which is that the data of relevance to this review is descriptive and not subject to robust analysis. Control site was substantially different in SES profile, making comparison between intervention and control in terms of PA subject to important biases. There is a lack of detail about potential contamination of other interventions occurring in the intervention sites. | Low |
| Rose, 2022  Australia | A before and after study with limitations to the outcome measures and data collection. Follow up at 6 months was short, and one might expect the nature of the intervention would take longer to 'embed'. Some items unclear, which makes overall risk of bias for this study unclear. | Low |
